# Transcriptional Active Parvovirus B19 Infection Predicts Adverse Long-Term Outcome in Patients with Non-Ischemic Cardiomyopathy

**DOI:** 10.3390/biomedicines9121898

**Published:** 2021-12-14

**Authors:** Felicitas Escher, Ganna Aleshcheva, Heiko Pietsch, Christian Baumeier, Ulrich M. Gross, Benedikt Norbert Schrage, Dirk Westermann, Claus-Thomas Bock, Heinz-Peter Schultheiss

**Affiliations:** 1 Institute of Cardiac Diagnostics and Therapy, IKDT GmbH, 12203 Berlin, Germany; ganna.aleshcheva@ikdt.de (G.A.); heiko.pietsch@charite.de (H.P.); christian.baumeier@ikdt.de (C.B.); ugross@zedat.fu-berlin.de (U.M.G.); heinz-peter.schultheiss@ikdt.de (H.-P.S.); 2 Department of Internal Medicine and Cardiology, Campus Virchow-Klinikum, Charité-Universitaetsmedizin Berlin, Corporate Member of Freie Universitaet Berlin and Humboldt-Universitaet zu Berlin, 13353 Berlin, Germany; 3 DZHK (German Centre for Cardiovascular Research), Partner Site Berlin, 13353 Berlin, Germany; 4 Department of Cardiology, University Heart and Vascular Center Hamburg, 20246 Hamburg, Germany; b.schrage@uke.de (B.N.S.); d.westermann@uke.de (D.W.); 5 DZHK (German Center for Cardiovascular Research), Partner Site Hamburg/Lübeck/Kiel, 20246 Hamburg, Germany; 6 Department of Infectious Diseases, Division of Viral Gastroenteritis and Hepatitis Pathogens and Enteroviruses, Robert Koch Institute, 13353 Berlin, Germany; BockC@rki.de; 7 Institute of Tropical Medicine, University of Tuebingen, 72074 Tuebingen, Germany

**Keywords:** parvovirus B19, transcriptional activity, dilated inflammatory cardiomyopathy, long-term outcome

## Abstract

Parvovirus B19 (B19V) is the predominant cardiotropic virus currently found in endomyocardial biopsies (EMBs). However, direct evidence showing a causal relationship between B19V and progression of inflammatory cardiomyopathy are still missing. The aim of this study was to analyze the impact of transcriptionally active cardiotropic B19V infection determined by viral RNA expression upon long-term outcomes in a large cohort of adult patients with non-ischemic cardiomyopathy in a retrospective analysis from a prospective observational cohort. In total, the analyzed study group comprised 871 consecutive B19V-positive patients (mean age 50.0 ± 15.0 years) with non-ischemic cardiomyopathy who underwent EMB. B19V-positivity was ascertained by routine diagnosis of viral genomes in EMBs. Molecular analysis of EMB revealed positive B19V transcriptional activity in *n* = 165 patients (18.9%). Primary endpoint was all-cause mortality in the overall cohort. The patients were followed up to 60 months. On the Cox regression analysis, B19V transcriptional activity was predictive of a worse prognosis compared to those without actively replicating B19V (*p* = 0.01). Moreover, multivariable analysis revealed transcriptional active B19V combined with inflammation [hazard ratio 4.013, 95% confidence interval 1.515–10.629 (*p* = 0.005)] as the strongest predictor of impaired survival even after adjustment for age and baseline LVEF (*p* = 0.005) and independently of viral load. The study demonstrates for the first time the pathogenic clinical importance of B19V with transcriptional activity in a large cohort of patients. Transcriptionally active B19V infection is an unfavourable prognostic trigger of adverse outcome. Our findings are of high clinical relevance, indicating that advanced diagnostic differentiation of B19V positive patients is of high prognostic importance.

## 1. Introduction

Viral infection of the heart can cause myocarditis and dilated inflammatory cardiomyopathy (DCMi) [1,2]. Following infection, patients may develop a chronic autoimmune inflammatory reaction [3,4,5]. Clinical outcome is determined by the interplay between virulence and host immune response, and may range from subclinical disease to congestive heart failure [6]. Enterovirus- and adenovirus persistence is proven to be associated with an increased mortality [7]. In recent decades, a shift has been observed to parvovirus B19 (B19V), a single-stranded DNA virus and member of the Parvoviridae family, genus Erythroparvovirus [8], as the most frequently found cardiotropic virus in endomyocardial biopsies (EMBs) [9,10]. Despite this common detection of B19V in EMBs, prognostic relevance of B19V prevalence has been denied so far [11]. Previous clinical studies suggested that B19V presence determined by viral genomes is an unspecific bystander and may have no prognostic value in myocarditis and DCMi [12]. However, primarily experimental data suggest a relevant effect of B19V on the development of myocarditis and DCMi [13,14].

Due to the virus receptor distribution, B19V persist lifelong within bone marrow precursor cells, and are causative in the vascular endothelium [15], leading to persistent B19V infection [16,17,18]. It is known that endothelial cells represent specific targets in B19V-associated myocarditis [19]. As the B19V infection of endothelial cells is associated with a symptomatic endothelial dysfunction [17,20,21], it has been reported with atherosclerosis, vasospasms, and progression of coronary vasculopathy, limiting organ transplant survival [14,22,23,24,25]. Thus, a causal relationship between parvovirus B19V-related infection of vascular endothelial cells and inflammation can be suspected [26].

Assessing the replicative status by measuring transcriptional activity of B19V is the most accurate way to determine active B19V infection in the heart [27], as the presence of B19V genome in the heart itself can indicate a latent virus without replicative activity [10].

B19V with transcriptional activity is expected to cause cardiac inflammation, damage and dysfunction, since altered cardiac gene expression has been demonstrated in patients with transcriptional activity in comparison to control patients and myocarditis patients with latent B19V [28].

In order to gain more specific insight into the pathophysiological significance of B19V infection in the development of DCMi and more detailed insights into the long-term effects of B19V with or without transcriptional activity, we conducted a retrospective study of a prospective large observational cohort of adult patients who had underwent EMB with proof of B19V-positivity, following them for up to 60 months and comparing their outcomes with respect to EMB analysis. The aim of this study was to analyze for the first time the prognostic role of transcriptional active B19V infection on long-term outcomes.

## 2. Materials and Methods

### 2.1. Total Study Cohort

The analyzed group comprised of 871 B19V-positive patients with clinical evidence of symptomatic heart failure underwent an EMB for clear diagnosis [29,30]. The patients included in this study were evaluated by means of extensive EMB diagnostics. The diagnosis of B19V positivity was made by B19V-specific nested PCR of viral genomes. Coronary artery disease was excluded angiographically in all patients prior to EMB. The patients complained about symptoms of heart failure with fatigue, reduced physical capacity or dyspnea on exertion, and cardiac dysfunction. 

Patients with myocardial virus infections other than B19V or coinfections such as enteroviruses, adenoviruses, or herpesviruses were excluded from this study [31]. Patients with other co-morbidities and known cardiac involvement were also excluded. Active myocarditis and storage diseases (amyloid, lime, iron) have been excluded by histological staining using Azan, hematoxylin eosin, EvG, and PAS.

All diagnostic procedures and evaluation were obtained using standardized protocols and questionnaires, respectively [32]. The median time from debut of first symptoms to diagnostic biopsy was six months. Indication of EMB was based on heart failure symptoms and reduced left ventricular ejection fraction (LVEF), resulting in the suspected diagnosis of chronic myocarditis/inflammatory cardiomyopathy [33].

The study endpoint was time to all-cause death. Occurrence of the endpoint (death) was determined through direct knowledge of the patient’s status, contact with family members, or inquiries at the registration office. The overall observation period was at mean 11.91 months (96% CI of mean 10.7–13.0 months). 

In a subgroup analysis of *n* = 222 patients, an additional LVEF measurement was taken after a follow-up period mean 21.4 months (96% CI of mean 19.1–23.6 months) and analyzed. LVEF baseline and at follow-up was determined by echocardiography. LVEF deterioration has been defined as Δ-LVEF, i.e., the difference between follow-up and baseline LVEF ≥−5%. Lack of improvement of LV function was determined as consistent LV dysfunction <35% at baseline and follow-up. 

### 2.2. Analysis of EMB

#### 2.2.1. Analysis of Viral Nucleic Acids in EMB, Genomic DNA Isolation from EMBs

EMB were analyzed in the CAP-accredited laboratory IKDT (Institute for Cardiac Diagnostic and Therapy Berlin, Germany) by molecular workup: Genomic DNA from RNAlater (Ambion, Austin, Texas, USA) fixed EMBs was extracted by Puregene Mousetail Kit (Gentra, Minneapolis, Minnesota, USA). After isolation the amount of isolated DNA was quantified by a specialised Quantifiler TaqMan assay (Applied Biosystems, Darmstadt, Germany), in order to calculate and standardize estimation of the virus load in small EMBs (viral genomes per µg of isolated human genomic DNA) [9].

#### 2.2.2. Detection of Viral Genomes in EMBs by Nested-PCR and Sequencing

Nested polymerase chain reaction (n-PCR) and reverse transcriptase (RT)-PCR for qualitative detection of B19V genome sequences in material extracted from EMB was applied by gel-electrophoresis. Detection of B19V DNA by nPCR was performed as described previously [9,34].

#### 2.2.3. Measurement of Viral DNA Load by Quantitative Real-Time PCR (TaqMan QPCR)

Subsequent calculation of viral DNA load was performed by ratio of estimated viral genome copy number in TaqMan assay to amount of incorporated human DNA amount measured by Quantifiler TaqMan assay (Applied Biosystems, Darmstadt, Germany) [28].

#### 2.2.4. RNA Isolation, Reverse Transcription (RT) and TaqMan QPCR for Measurement of Viral Transcripts

Total RNA was isolated from endomyocardial biopsies using Trizol reagent (Invitrogen, Karlsruhe, Germany), treated with DNAse (PeqLab, Erlangen, Germany) to remove any traces of genomic DNA and reverse-transcribed to cDNA with the High capacity Kit (Applied Biosystems, Darmstadt, Germany) using random hexamers.

The copy numbers of viral transcripts in cDNA were determined by real-time PCR using TaqMan Universal PCR master mix (Applied Biosystems, Darmstadt, Germany) as described previously [28] (Table 1). 

#### 2.2.5. Histological and Immunohistochemical Staining for Assessment of Inflammation

Histology was developed by hematoxylin eosin staining in light microscopy. Immunohistochemistry was used for the characterization of inflammatory infiltrates and carried out on RNA-later fixation. Stainings were quantified by digital image analysis as described previously [35]. Intramyocardial inflammation was assigned according to the ESC statement [33] by CD3+ t-lymphocytes/mm^2^ (Dako, Glostrup, Denmark), CD11a+/LFA-1+ lymphocytes/mm^2^ (Immuno Tools, Friesoythe, Germany), CD11b+/Mac-1+ macrophages/mm^2^ (ImmunoTools, Friesoythe, Germany), and CD45R0+ T memory cells (Dako, Glostrup, Denmark). Stainings were quantified by digital image analysis as described previously [35].

### 2.3. Ethical Approval

The study was performed within the CRC Transregio 19 (NCT02970227) approved by the by Institutional Ethics Committee (Charité Berlin). Informed written consent was obtained from each study patient and the protocol conforms to the ethical guidelines of the 1975 Declaration of Helsinki.

### 2.4. Statistical Analysis

All analyses were performed using R 3.5.3 and GraphPad Prism 9. Qualitative data were compared conducting the χ2-test. The Student’s *t*-test was used to analyze continuous variables. Results are presented as mean ± standard deviation or given as median value (10th, 90th percentile). Survival curves were generated according to the Kaplan-Meier method and compared with the log-rank statistic. The initial time point for each survival analysis was the date of myocardial biopsy. To analyze the association between transcriptional activity and inflammation with all-cause mortality, multivariable Cox regression models were fitted with adjustments for age and baseline-LVEF. A probability value of *p* < 0.05 was considered statistically significant.

## 3. Results

### 3.1. Total Patient Cohort

The study group was comprised of 871 consecutive B19V-positive patients who underwent EMB performed at first admission.

The baseline data of patient characteristics and EMB analyses are given in Table 2. The cohort comprised EMBs from *n* = 555 (63.7%) male and *n* = 316 (36.2%) female patients at a mean age of 50.0 ± 15.0 years. The mean LVEF was 48.6 ± 20.0 % at baseline. The patient complaints for symptoms of heart failure included reduced physical capacity (85.1%) and NYHA II 54.37%/NYHA III 31.70%/NYHA IV 4.5%.

In the molecular biological analysis of 871 EMBs, B19V-positive transcriptional activity was detected in 165 patients, (18.9%) preferably men (64.8%) of the mean age of 50.0 ± 15.0 years old with mean LVEF of 44.9.0 ± 19.2% at baseline (Table 2).

**Figure 1:**
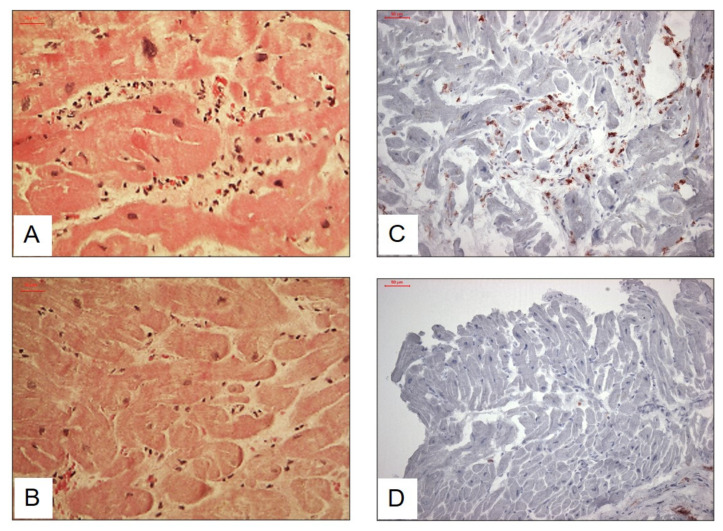
Representative images if (Immuno-) histopathological findings from endomycardial biopsies of B19V positive patients. (**A**). H&E staining. Patient with B19V with transcriptional activity and inflammation. Inflammatory cellular infiltrates in between myocytes preferentially in the neighborhood of small vessels. Nuclei and cytoplasm of myocytes vary in diameter. (**B**). H&E staining. Patient with B19 V without transcriptional activity without inflammation. Varying diameter of myocytes. (**C**). Immunohistological staining of increased CD3 positive T lymphocytes near small vessels between myocytes with some variation of diameter in a patient with B19V with transcriptional activity. (**D**). Immunohistological staining of low CD3 positive T lymphocytes infiltration in a patient with B19V without transcriptional activity. Scale bars: 50 µm.

According to the immunohistochemical analyses, intramyocardial inflammation was detected in 436 patients (50.1%). Quantification of infiltrative cells did not yield a significant difference regarding B19V-RNA^+^ vs. B19V-RNA^−^ in the total cohort. Intramyocardial inflammatory infiltration was preferentially found in the neighborhood of small vessels (Figure 1).

### 3.2. Predictors of Outcome

#### 3.2.1. B19V Viral Load 

**Figure 2:**
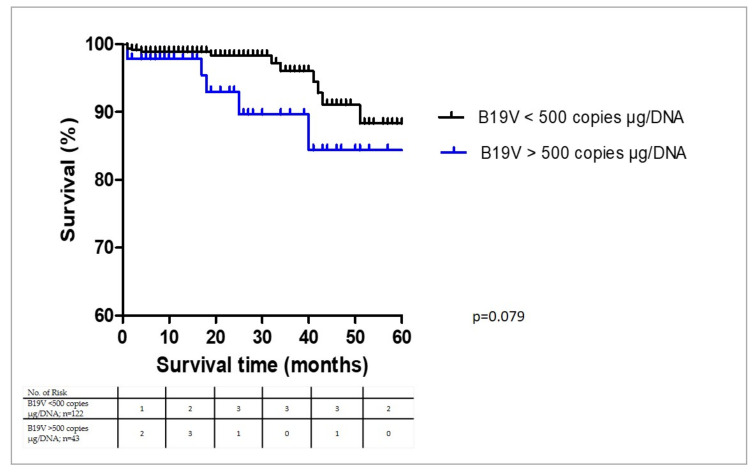
Kaplan-Meier plots in total patient cohort for all-cause mortality. Five-year outcome of B19V-positive patients with copy numbers above or below 500 copies/μg isolated DNA.

As the B19V copy numbers above 500 copies/μg isolated DNA have been considered as being clinically relevant [14], we determined a quantitative measurement of the B19V DNA copy numbers. Neither the mean virus loads nor the frequency of patients with viral copy numbers below or above 500 copies/μg isolated DNA (Figure 2) were significantly different. The same holds true if the threshold was set at 1000 copies (*p* = 0.602) or 2000 copies (*p* = 0.795). This indicates that the virus load alone has no direct impact on the outcome.

**Figure 3:**
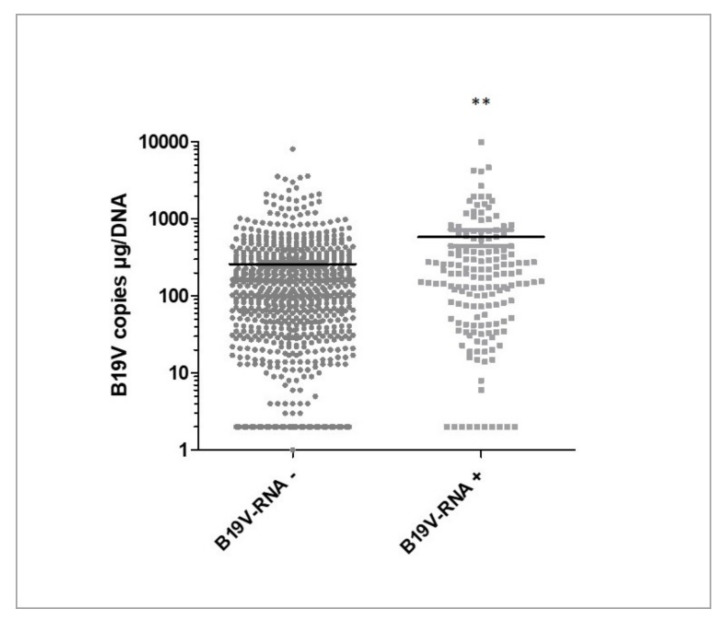
Box blot showing high statistical difference between B19V copy numbers in B19V-RNA^+^ (*n* = 165) vs. B19V-RNA^−^ (*n* = 706) (** *p* = 0.006). B19V = parvovirus B19; B19V-RNA^+^ = B19V with transcriptional activity; B19V-RNA^−^ = B19V without transcriptional activity.

As a next step, B19V transcriptional activity and copy numbers were evaluated. B19V DNA copy numbers were significantly elevated in patients with transcriptional activity (B19V-RNA^+^) (Figure 3), however, there was no correlation between DNA load and extent of viral transcription (mRNA copy number) *p* = 0.3.

#### 3.2.2. B19V Transcriptional Activity 

**Figure 4:**
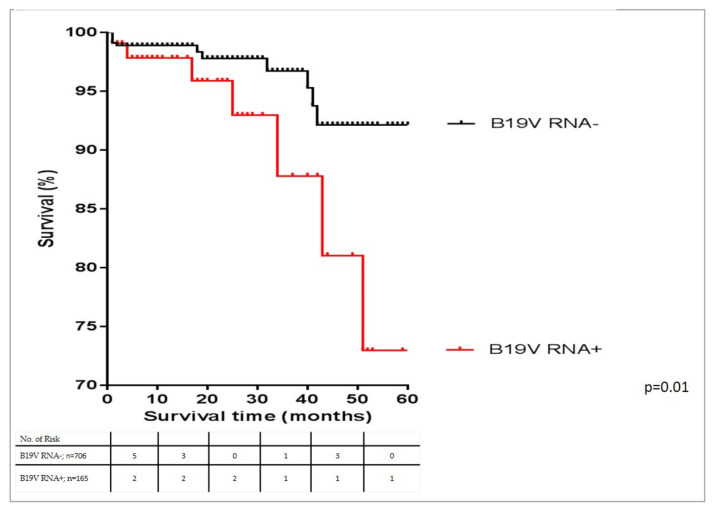
Kaplan-Meier plots in total patient cohort for all-cause mortality. Five-year mortality of B19V-positive patients in dependence of B19V and transcriptional activity. The mortality rate was higher in patients with transcriptional activity (*n* = 165) compared to those without transcriptional activity (*n* = 706).

After examining of transcriptional activity in the patient cohort as the main result of our study on the Kaplan–Meier curve analysis, B19V transcriptional activity was predictive of a worse prognosis, compared to those without actively replicating B19V (*p* = 0.01 by the log-rank test) (Figure 4).

We could confirm via the Cox regression analysis a significant difference in mortality rate in patients negative for B19V transcriptional activity with vs. without inflammation (*p* = 0.03). Corresponding to our main statement, the cohort of patients with B19V-RNA^+^ with inflammation exhibited a significantly worse prognosis in contrast to patients with B19V-RNA^−^ with inflammation in the total cohort (*p* = 0.04).

Moreover, the majority of fatal causalities was found in patients with actively replicated B19V and inflammation. In the multivariable Cox regression analysis, transcriptionally active B19V combined with inflammation [hazard ratio 4.013, 95% confidence interval 1.515–10.629 (*p* = 0.005)] was the strongest predictor and was associated with higher mortality as compared to the absence of transcriptional activity and the absence of inflammation; even after adjustment for age and baseline LVEF (Table 3).

#### 3.2.3. Subgroup Analysis

**Figure 5:**
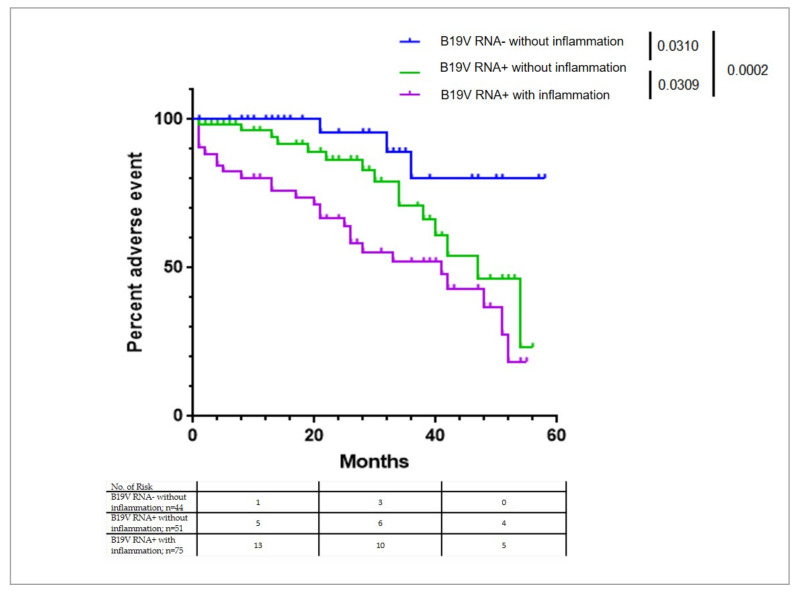
Kaplan-Meier plots for composite end-point LV deterioration and all-cause mortality. In a subgroup analysis of *n* = 222 patients with haemodynamic measurements after a long follow-up period, a Cox analysis confirmed significant association between B19V transcriptional activity and LVEF deterioration, lack of improvement of LV function, and higher mortality even without inflammation in EMB (*p* = 0.03).

In a subgroup analysis of *n* = 222 patients with haemodynamic measurements after a long follow-up period, a Cox analysis of composite end-point including LVEF deterioration, lack of improvement of LV function, and all-cause mortality confirmed significant association between B19V transcriptional activity and the combined end-point (0.0002). Interestingly, this subgroup analysis also showed a significant difference in the groups with and without transcriptional activity even without inflammation in EMB (*p* = 0.03 by the log-rank test) (Table 4, Figure 5).

## 4. Discussion

In this study, we demonstrated for the first time in a large cohort of patients with B19V infection in EMB that viral B19V genomes in the presence of replicative intermediates (viral RNAs) are related to adverse long-term clinical outcomes. This is the first demonstration of the clinical pathophysiological significance of B19V with transcriptional activity as contrasted with latent B19V.

B19V as the predominant pathogen currently detected in EMBs of patients with DCMi is underestimated from a clinical perspective. Its wide circulation generally leads to a diminished appreciation of its pathogenetic potential. Over the last decade, B19V has frequently been linked to the pathogenesis of DCMi and its progression towards dilated cardiomyopathy (DCM) [36]. Nevertheless, direct evidence showing a causal relationship between B19V cardiac presence and disease progression of B19V-associated DCMi were missing until now. 

Due to our large collective of adult B19V positive patients, in this study we were able to demonstrate for the first time that B19V has a clinical relevance associated with evidence of transcriptional activity in EMBs. To gain more information on the clinical relevance of B19V with and without viral RNA transcriptional activity, we followed B19V-positive patients with reduced LVEF up to 60 months. On Cox regression analysis, B19V transcriptional activity was predictive of a worse prognosis, compared to those without actively replicating B19V. Moreover, this effect is exacerbated in the presence of inflammation. In the multivariable analysis, transcriptional active B19V combined with inflammation proved to be the strongest predictor of impaired survival. In addition, we conducted a subgroup Cox analysis with composite end-point confirmed significant association between B19V transcriptional activity and LV deterioration or lack of improvement of LV function even without inflammation in EMB. 

Due to elevated B19V copy numbers in patients with DCM/DCMi in comparison to controls, 500 virus copies have been considered as a clinically important threshold value [14]. In inflamed and uninflamed hearts, copy numbers of the B19V genome and elevated copy numbers above 500 copies/µg isolated DNA were neither different nor associated with an impaired clinical outcome. This is in line with the general clinical experience that the load of B19V per se does not correlate with the clinical course or complaints of patients with heart failure [21].

Interestingly, and in contrast to other cardiotropic viruses, B19V exclusively targets vascular endothelial cells [20,37]. If infected cells are injured by a lytic virus infection or by the antiviral immune response, replaceable endothelial cells but not irreplaceable cardiomyocytes are destroyed. In addition, myocardial inflammation in response to viral infection was found to be associated with increased endothelial expression of the human leucocyte antigen system and adhesion molecules [19,38]. The presence of B19V remained a single independent predictor for reduced cardiac vascular density in patients with cardiomyopathy. This result demonstrates the importance of B19V infection for reduced coronary blood flow leading to ischemia and endothelial dysfunction. This is an explanation as to why acute endothelial cell B19V-infection is associated with a cardiac microvascular impairment mimicking myocardial infarction [39,40,41]. It would also be in line with reports on progressive alterations of the vasculature and reduced survival of transplanted hearts in B19V-positive children or improvement of endothelial cell function upon antiviral treatment strategies [20,22,42]. In contrast to enteroviral and adenoviral infections which are acquired at any age, B19V permanently resides in the bone marrow and vascular endothelial cells after primary infection [43]. It is caused by endogenous B19V-infected bone marrow derived endothelia progenitor cells which are released into the bloodstream during vascular repair [16,20]. In this respect, the EMB proof of B19V in adults constitutes a completely distinct disease entity.

The importance of testing viral presence for further treatment has been shown in a retrospective analysis by Frustaci in which viral persistence was associated with detrimental outcomes in patients with myocarditis receiving immunosuppressive therapy [44]. Proofing of transcriptional activity of B19V is therefore mandatory to differentiate between latent and active viral persistence and avoiding potential incorrect therapeutic decisions.

Primary antiviral therapeutic options already exist. Recently, we were able to show that clinical symptoms in patients with enterovirus and adenovirus genomes improved during antiviral treatment with interferon-β [45]. Moreover, suppression of B19V transcriptional activity by nucleoside analogue telbivudine treatment improved hemodynamic and clinical outcomes significantly [46]. In- vitro data provided an anti-apoptotic effect of this nucleoside analogue through normalization of BIRC3 levels in B19V-induced apoptosis, and thereby protected cells from B19V damage [47].

### Limitation of the Study

The major limitation of this study is the lack of randomization. This was a retrospective analysis of data from a prospective observational cohort and, as such, the possible effect of selection bias cannot be denied. The cohort was only adjusted to B19V-positive patients with or without detectable replicative intermediates of B19V confirmed by measurement of B19V RNA transcripts. In this design, unmeasured confounding cannot be ruled out.

## 5. Conclusions

Among patients with B19V genomes in EMB, in this study we were able to demonstrate for the first time that B19V with transcriptional activity is clinically relevant. Transcriptionally active B19V is associated with progressive cardiac dysfunction and impaired survival.

Compentency in medical knowledge: Our study has high and immediate clinical impact, since B19V is the most frequently found cardiotropic virus in EMBs. Testing of replicative status of B19V is a prerequisite for further therapeutic decisions. The findings reported here highlight the importance of an advanced diagnostic approach with inclusion of B19V transcriptional activity into the routine evaluation of EMBs, especially if inflammation is detected. This is of particular importance because initiation of an immunosuppressive therapy leads to a significantly worse prognosis in the case of an additional active viral infection.

Translational outlook: Comprehensive, standardized diagnostic differentiation of endomyocardial B19V infections with biological and prognostic relevance (including viral replication and ongoing inflammation) seems to be the key approach for both an updated diagnostic classification of B19V associated viral cardiomyopathy and a more meaningful selection of candidates for future innovative anti-viral immunomodulatory treatment strategies and randomized studies, which are urgently needed.

## Figures and Tables

**Table 1:** Primer sequences used for detection of B19V.

Primer/Probe Name	Nucleotide Sequence (5′–3′)
NS1-FW	TCCCTGGAATWAATGCAGATGC
NS1-RV	CACTGCTGCTGAYACTGGTGTCT
NS1-probe	6FAM-ACCTCCAAACCACCCCAATTGTCACA-TAMRA
VP1-FW	TGTAAGATGCAGCCCTGACATG
VP1-RV	GAGGGCATCTGCATTAATTCCA
VP1-probe	6FAM-TGGTGTAATGCACAAAGCTGG-TAMRA

Note: B19V = parvovirus B19; FW = forward; NS1 = nonstructural B19 protein NS1; RV = reverse; VP1 = structural B19V protein VP1.

**Table 2:** Clinical characteristics and EMB analysis at baseline with and without transcriptional activity in total patient cohort.

Clinical Characteristics	All Patients	With Replicative Intermediates (B19V-RNA^+^)	Without Replicative Intermediates (B19V-RNA^−^)
Number of patients, *n* (%)	871 (100)	165 (18.9)	706 (81.1)
Sex, *n* (%)	555 (63.7) male 316 (36.2) female	107 (64.8) male 58 (35.1) female	448 (63.4) male 258 (36.5) female
Age, mean ± SD (years)	50.0 ± 15.0	50.0 ± 15.0	49.0 ± 15.0
LVEF, mean ± SD (%)	48.6 ± 20.0	44.90 ± 19.2	49.4 ± 18.6 ^a^
LVEDD, mean ± SD (mm)	56.7 ± 9.1	58.0 ± 9.6	55.8 ± 9.7 ^a^
LVESD, mean ± SD (mm)	40.4 ± 12.3	42.4 ± 13.2	41.0 ± 12.7
infection preceding onset of symptoms <12 weeks (%), *n* = 429	52.5	56.0	50.5
- flue like (%)	38.9	45.4	35.4
- pneumonia (%)	8.4	6.6	9.4
- gastrointestinal (%)	1.6	0.7	2.2
- other (%)	3.5	3.3	3.6
Complaints at baseline biopsy			
Reduced physical capacity (%)	85.1	90.5	82.1
NYHA II/III/IV (%)	54.3/31.7/4.5	56.4/29.1/5.1	50.1/34.2/3.7
Angina at rest (%)	22.4	26.7	20.1
Angina on exertion (%)	40.1	41.0	39.2
Palpitation (%)	8.5	6.8	10.8
Systolic blood pressure, mean ± SD (mmHg)	118 ± 18	115 ± 18	117 ± 17
Diastolic blood pressure, mean ± SD (mmHg)	74 ± 11	74 ± 11	74 ± 1
Peripheral edema (%)	29.4	30.9	28.6
Medication, *n*			
- β-blockers	48.7	47.9	49.1
- ACE inhibitors/ARB	71.8	72.2	71.9
- diuretic agents	52.2	50.1	53.1
Biopsy (inflammatory cell counts)			
CD3^+^, mean ± SD (cells/mm^2^)	7.4 ± 12.0	7.2 ± 6.9	7.5 ± 13.0
CD45RO^+^, mean ± SD (cells/mm^2^)	21.5 ± 21.8	23.8 ± 19.2	21.0 ± 22.4
LFA-1^+^, mean ± SD (cells/mm^2^)	17.4 ± 25.0	16.8 ± 15.2	17.5 ± 26.8
Mac-1^+^, mean ± SD (cells/mm^2^)	34.8 ± 28.0	35.3 ± 24.9	34.7 ± 28.7

Note: ACE inhibitor = angiotensin-converting-enzyme inhibitor; ARB = angiotensin receptor blocker; B19V = Parvovirus B19; CD3 = t cells; CD45R0 = t memory cells; LFA-1 = Lymphocyte function-associated antigen 1; LVEDD = left ventricular end-diastolic diameter, LVEF = left ventricular ejection fraction; LVESD = left ventricular end-systolic diameter; MAC-1 = Macrophage-1 antigen.; NYHA = New York Heart Association. The data are presented as mean ± standard deviation (SD), as %, and as No. of subjects. ^a^ Significantly different; B19V-RNA^+^ vs. B19V-RNA^−^.

**Table 3:** Multivariable Cox regression analysis of total patient cohort.

	Unadjusted Cox Model			Adjusted Cox Model		
Group	HR	95%CI	*p*-Value	HR	95%CI	*p*-Value
B19V-RNA^+^ without inflammation vs. B19V-RNA^−^ without inflammation	1.020	0.223–4.658	0.980	1.004	0.219–4.559	0.996
B19V-RNA^+^ with inflammation vs. B19V-RNA^−^ without inflammation	3.239	1.223–8.575	0.018	4.013	1.515–10.629	0.005

Note: Cox regression model in total patient cohort with time to death as the dependent variable and B19V replication/inflammation as the independent variable, with additional adjustment for age and ejection fraction at baseline.

**Table 4:** Subgroup analysis.

Clinical Characteristics	With Replicative Intermediates (B19V-RNA^+^)	Without Replicative Intermediates (B19V-RNA^−^)
Number of patients, *n* (%)	126	96
Age, mean ± SD (years)	48.1 ± 16.4	48.3 ± 13.2
LVEF, mean baseline ± SD (%)	45.5 ± 18.2	48.6 ± 19.1 ^a^
LVEF, mean follow-up ± SD (%)	52.0 ± 17.2 ^b^	56.8 ± 15.3 ^ab^
LVEF recovery (%)	38.0	52.0 ^a^
LVEDD, mean baseline ± SD (mm)	55.8 ± 9.5	56.2 ± 10.4
LVEDD, mean follow-up ± SD (mm)	58.1 ± 9.0	55.3 ± 10.2 ^a^
LVESD, mean baseline ± SD (mm)	41.4 ± 12.9	41.6 ± 14.7
LVESD, mean follow-up ± SD (mm)	44.9 ± 12.0	40.4 ± 14.0
Medication, %		
- β-blockers	47.5	49.2
- ACE inhibitors/ARB	73.2	72.3
- diuretic agents	51.1	53.1
Biopsy (inflammatory cell counts)		
CD3^+^, mean ± SD (cells/mm^2^)	10.3 ± 14	9.9 ± 21.3
CD45R0^+^, mean ± SD (cells/mm^2^)	28.5 ± 29.7	26.8 ± 72.5
LFA-1^+^, mean ± SD (cells/mm^2^)	23.6 ± 26.6	29.6 ± 118
Mac-1^+^, mean ± SD (cells/mm^2^)	43.1 ± 33.9	48.6 ± 123.3

Note: ACE inhibitor = angiotensin-converting-enzyme inhibitor; ARB = angiotensin receptor blocker; B19V = parvovirus B19; LVEDD = left ventricular end-diastolic diameter, LVEF = left ventricular ejection fraction; LVESD = left ventricular end-systolic diameter; CD3 = t cells; CD45R0 = t memory cells; LFA-1 = Lymphocyte function-associated antigen 1; MAC-1 = Macrophage-1 antigen. The data are presented as mean + standard deviation (SD), as %, and as No. of subjects. ^a^ Significantly different; B19V-RNA^+^ vs. B19V-RNA^−^. ^b^ Significantly different; baseline vs. follow-up.

## Data Availability

The data presented in this study are available on request from the corresponding author.
